# Compare 30-minute automated office blood pressure vs 24-hour ambulatory blood pressure monitoring: A prospective study

**DOI:** 10.1371/journal.pone.0360957

**Published:** 2026-11-02

**Authors:** Ai Cao My Bui, Quynh Tran Nhat Nguyen, Anh Ngoc Minh Bui, Minh Vu Phuc Ha, Nhan Phu Nguyen, Thy Ha Anh Nguyen, Nhan Quoc Dang, Phuong Minh Tran, Tu Vu Kha, Nhu Dong Phuong Nguyen, Liem Diep Bao Le, Huy Hoai Tran, Dung The Bui, Nghia Phu Nguyen

**Affiliations:** 1 School of Medicine, University of Medicine and Pharmacy at Ho Chi Minh City, Ho Chi Minh City, Vietnam; 2 Department of Interventional Cardiology, University Medical Center Ho Chi Minh City, Ho Chi Minh City, Vietnam; 3 Faculty of Public Health, University of Medicine and Pharmacy at Ho Chi Minh City, Ho Chi Minh City, Vietnam; 4 Department of Cardiology, University Medical Center Ho Chi Minh City, Ho Chi Minh City, Vietnam; University Medical Centre Maribor: Univerzitetni Klinicni Center Maribor, SLOVENIA

## Abstract

**Background:**

Ambulatory blood pressure monitoring (ABPM) is the reference standard for diagnosing hypertension (HTN), but its use in routine practice remains limited because of cost, availability, and patient burden. Automated office blood pressure (AOBP) has been proposed as a practical alternative, although its performance relative to ABPM remains uncertain.

**Objectives:**

To evaluate the agreement and diagnostic performance of 30-minute AOBP compared with 24-hour ABPM and to identify optimal cut-off values for detecting ambulatory hypertension.

**Methods:**

In this prospective cohort study, 79 adults undergoing ABPM also underwent 30-minute AOBP measurement with automated readings every 5 minutes. The mean of the final six measurements was analyzed. Agreement was assessed using paired comparisons and Bland–Altman analysis. Diagnostic performance using receiver operating characteristic (ROC) analysis and agreement statistics.

**Results:**

Thirty-minute AOBP values were consistently higher than ABPM measurements. Mean differences were +6.59/ + 7.27 mmHg for systolic/diastolic BP versus 24-hour ABPM and +3.73/ + 5.16 mmHg versus daytime ABPM (all p < 0.001). Bland–Altman analysis showed acceptable group-level agreement but wide limits of agreement. Sensitivity ranged from 84% to 96% and specificity from 28% to 79%. ROC analysis demonstrated good discrimination (AUC 0.77–0.87). The highest agreement with overall ABPM occurred at a cut-off of ≥140/84 mmHg (κ = 0.624).

**Conclusions:**

Thirty-minute AOBP showed good discrimination for elevated ambulatory blood pressure but systematically overestimated BP relative to ABPM. It may serve as a practical screening tool, although normal AOBP values cannot reliably exclude ambulatory HTN and confirmation with 24-hour ABPM remains necessary.

**Central Illustration:**

Diagnostic Performance of 30-Minute Automated Office Blood Pressure Compared With 24-Hour Ambulatory Monitoring

## Introduction

Hypertension (HTN) is the most common chronic condition in clinical management and is a major risk factor for cardiovascular events [1–4]. For that reason, accurate diagnosis is critical for instituting effective treatment and mitigating the risk of complications [5,6]. However, accurate measurement of blood pressure (BP) is challenging due to several factors, including physiological variability, psychological triggers, non-standardized measurement techniques, and observer bias. This often leads to the misdiagnosis of white-coat hypertension (WCH) or the underdetection of masked hypertension (MH) [7–9].

Ambulatory blood pressure monitoring (ABPM) is an out-of-office monitoring method widely considered the gold standard for BP measurement [10]. ABPM entails a patient wearing a device that continuously records BP over 24–48 hours. The device is programmed to measure the patient’s BP every 15–30 minutes while awake and 30–60 minutes while asleep, which captures the full range of BP variability during daily activities. ABPM offers a more exhaustive and precise representation of a patient’s actual blood pressure compared to isolated clinical measurements. Moreover, it facilitates the identification of irregular clinical signs, including nocturnal HTN and a non-dipping pattern, characterized by a failure of BP to decline during sleep [11–14].

Beyond its diagnostic role, clinical guidelines emphasize the utility of ABPM in various scenarios beyond suspected BP abnormalities. These include investigating suspected postprandial or orthostatic hypotension, evaluating symptoms suggestive of hypotension during treatment, and assessing resistant HTN. Furthermore, ABPM is essential for evaluating nocturnal BP profiles and dipping patterns in high-risk populations, such as those with obstructive sleep apnea (OSA), chronic kidney disease (CKD), diabetes mellitus (DM), endocrine HTN, or autonomic dysfunction [15,16]. Despite having established clinical value, ABPM remains an underutilised tool, as its implementation in routine practice faces several challenges [11]. The requirement for 24-hour continuous monitoring often results in patient discomfort and disrupts both daily routines and sleep patterns [17,18].

As an alternative, 30-minute AOBP monitoring has been suggested because its results are comparable to daytime ABPM. This method involves multiple automated measurements taken over a short period in a clinical setting, aiming to mimic ambulatory results. In this protocol, the patient rests alone in a quiet space while wearing a compact automated BP monitor programmed to obtain measurements every 5 minutes for a total of 7 readings over 30 minutes. The definitive value is derived by averaging the recorded measurements, usually after discarding the initial one or two readings to enhance data reliability. This method helps mitigate stress-related factors during measurement, effectively replicating the physiological conditions of daily ambulatory monitoring. Preliminary evidence suggests that 30-minute AOBP may effectively eliminate the white-coat effect, providing a more accurate reflection of a patient’s true BP levels compared to conventional single-office measurements [19–23].

The research team hypothesised that, if 30-minute AOBP monitoring demonstrates diagnostic performance comparable to 24-hour ABPM, it could represent a more time-efficient approach for assessing BP in clinical practice. Relative to 24-hour ABPM, this abbreviated protocol may reduce patient burden and improve feasibility in routine HTN assessment and management. Such an approach may be particularly relevant in many Asian health systems, where high patient volumes, limited ABPM availability, and resource constraints can restrict the widespread use of 24-hour monitoring. However, its potential value depends on rigorous evaluation of diagnostic accuracy and clinical utility against the 24-hour ABPM reference standard. At present, substantial evidence gaps remain regarding its performance and implementation in real-world settings. Accordingly, the central research question is: Can 30-minute AOBP monitoring serve as an effective substitute for 24-hour ABPM? In addition, this study aimed to explore the optimal systolic blood pressure (SBP) and diastolic blood pressure (DBP) cut-off values for 30-minute AOBP in identifying HTN defined by 24-hour ABPM.

## Materials and methods

### Study design and setting

A prospective cohort study was conducted on patients at the Cardiovascular Internal Medicine Clinic of University Medical Center Ho Chi Minh City, Vietnam. Patients were prescribed 24-hour ABPM. Study population was collected between 01/11/2024 and 28/02/2025, using the convenience sampling method. This study was reported in accordance with the Strengthening the Reporting of Observational Studies in Epidemiology (STROBE) guidelines [24].

### Study population

Participants were eligible if they met all the following criteria: (1) aged ≥ 18 years and underwent 24-hour ABPM prescribed by a physician, (2) provided informed consent to participate in this study, (3) adhered to the ABPM protocol. Patients meeting any of the following criteria were excluded: (1) a history of atrial fibrillation, (2) upper arm circumference incompatible with the standard ABPM device used at the hospital, (3) acute medical conditions that required immediate care, (4) night-shift workers.

### Sample size, sampling, and data collection

The process is summarized in Fig 1. The minimum required sample size was calculated using the formula for estimating the difference between two means. Based on the study by van der Wel et al. (σ = 15 mmHg, δ = 5 mmHg), the required sample size was 95 patients [19]. A convenience sampling method was applied. Among the 99 patients initially screened, 20 were excluded because they did not meet the inclusion criteria (n = 5), had technical errors or incomplete data (n = 5), or declined to participate (n = 10), leaving 79 patients for the final analysis.

**Fig 1:**
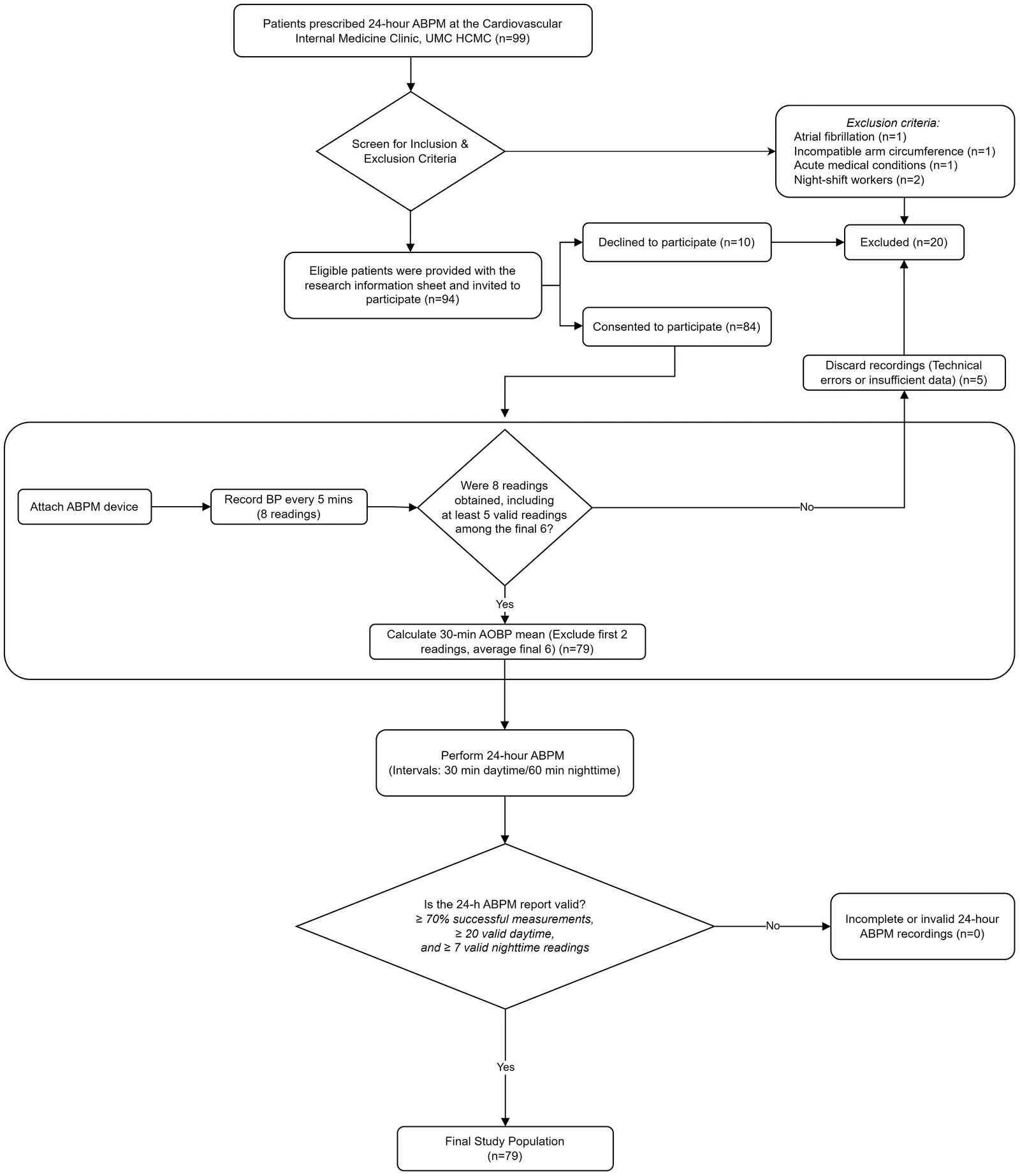
Participant selection and blood pressure measurement process.

(1) Before initiation of the 30-min AOBP measurement, the investigator approached the patient to provide detailed information about the study and obtain written informed consent. (2) The investigator then recorded the patients’ baseline characteristics, office BP measurements, ABPM indication, and other relevant clinical information using the structured questionnaire developed for this study. (3) Subsequently, the ABPM device was attached to the patient for BP monitoring. During the first 30 minutes, the device recorded BP every 5 minutes. The cutoff for 30-min AOBP was applied to the arm with higher office BP, provided that the inter-arm BP difference did not exceed 15 mmHg in 5 minutes in all 8 readings. During device setup, the first reading was considered a trial measurement. The second measurement took place when the 30-min period began. Results were recorded after 30 minutes. Recordings were discarded if technical errors occurred or if fewer than eight measurements were obtained. The first two readings were excluded, as previous studies have indicated that in sequential measurements, BP might decline significantly during the initial 10 minutes before stabilizing. The final 30-min AOBP value was calculated as the mean of the final six measurements. In situations where more than 1 of 6 readings was invalid, the 30-min AOBP data of that participant would be omitted.

Once the 30-min AOBP readings had been recorded, 24-hour ABPM was subsequently performed using the same device. The device was programmed to monitor BP every 30 minutes from 7:00–21:59 and every 60 minutes from 22:00–6:59 in the next morning. Prior to device placement, participants were instructed by a trained technician regarding adherence requirements during the monitoring period, including the following: (1) wear the device consecutively for 24 hours, and do not remove it without authorization, (2) during the 24-hour monitoring period, patients could continue performing daily activities while avoiding strenuous exercises, and keep the monitored arm extended and still during measurements, (3) if any abnormal symptom was present during measurement, press the button to mark the time, record the symptoms and their exact time of occurrence in a diary, which would be reviewed by the responsible physician upon device removal, (4) after 24 hours of monitoring, patients were scheduled to return to the clinic for device removal, (5) upon completion of monitoring, the recorded data were downloaded to a computer and a report was generated.

ABPM recordings were deemed valid for analysis if at least 70% of the total measurements obtained over the 24-h period were successful, including a minimum number of 20 valid daytime readings and 7 valid nighttime readings.

### Criteria used in this study

The following standardized criteria are employed in this study:

(1) Body Mass Index (BMI): BMI is classified according to the criteria established by the International Diabetes Institute (IDI) and the World Health Organization Western Pacific Region (WPRO) for Asian populations [25].
(2) Dyslipidemia: Dyslipidemia is defined based on the National Cholesterol Education Program – Adult Treatment Panel III (NCEP–ATP III) [26]. The patient is categorized as having dyslipidemia if they meet at least one of the following four criteria: triglycerides ≥ 200 mg/dL (≥ 2.26 mmol/L), low-density lipoprotein cholesterol (LDL-C) ≥ 160 mg/dL (≥ 4.14 mmol/L), total cholesterol ≥ 240 mg/dL (≥ 6.22 mmol/L), or high-density lipoprotein cholesterol (HDL-C) ≤ 40 mg/dL (≤ 1.04 mmol/L). Individuals with a previously documented medical diagnosis of dyslipidemia are also included in this group.
(3) DM: The diagnosis of DM is determined in accordance with the clinical diagnostic criteria provided by the American Diabetes Association (ADA) [27].

### Variable descriptions

Descriptive statistics:

- For continuous variables: described as mean value and standard deviation (if data is normally distributed), or the median value and interquartile range (if data is non-normally distributed).
- For categorical variables: presented as frequencies and percentages of each category. Pie charts were constructed to illustrate the proportional distribution of categorical variables across groups.

Inferential statistics: In this study, the primary exposure variables include: 30-min AOBP and 24-h ABPM. HTN by ABPM was defined according to established guideline thresholds as the presence of any of the following [9,15,28]: a daytime mean SBP ≥ 135 mmHg and/or DBP ≥ 85 mmHg; a nighttime mean SBP ≥ 120 mmHg and/or DBP ≥ 70 mmHg; or a 24-hour mean SBP ≥ 130 mmHg and/or DBP ≥ 80 mmHg. Because no guideline-based diagnostic threshold has been established for 30-minute AOBP, and previous studies have used a threshold of 135/85 mmHg in comparisons with daytime ABPM, we prespecified ≥130/80 mmHg as the initial threshold to correspond to the 24-hour ABPM threshold. Alternative cut-offs corresponding to 24-hour, daytime, and nighttime ABPM were subsequently explored using ROC analysis and were considered exploratory.

### Statistical methods

BP readings captured from the two measurement methods were tested using the paired Student’s t-test (if the data is normally distributed) and Wilcoxon (if the data is non-normally distributed), then visualized using Bland-Altman plots in order to compare the mean values of two methods. The Receiver operating characteristic (ROC) curve was employed to identify the optimal diagnostic cut-off value of the 30-min AOBP method. Logistic regression was performed to recognize factors associated with agreement between the 30-min AOBP and 24-h ABPM measurement methods. No missing values were present among the variables included in the final analyses. Data were stored using Microsoft Excel for Microsoft 365, Version 2608 and analyzed using R version 4.6.1 (R Foundation for Statistical Computing, Vienna, Austria).

### Ethical considerations

The study protocol was reviewed and approved by the Protocol Review Board of the Faculty of Medicine and the Biomedical Research Ethics Committee of the University of Medicine and Pharmacy at Ho Chi Minh City (Approval No. 2850/HDDD-DHYD; October 14, 2024). All participants provided written informed consent before enrollment, and the study was conducted in accordance with the principles of the Declaration of Helsinki.

### Results

#### Study population

A total of 79 participants were enrolled (Table 1), of whom 61% were male. The median age was 35 years (IQR: 26–58). None of the participants had a prior diagnosis of arterial hypertension or were receiving antihypertensive medication at the time of study inclusion. The mean BMI was 24.8 ± 3.8 kg/m^2^, and more than half of the participants were classified as overweight or obese. Dyslipidemia, hyperuricemia, and DM were reported in 54%, 22%, and 14% of participants, respectively. The median travel time to the hospital was 0.58 hours (IQR: 0.33–1.00). Most participants traveled by motorbike, underwent monitoring in the morning, and stayed at home during the monitoring period.

**Table 1:** Baseline characteristics of the study population (n = 79).

Characteristic	n (%)
**Demographics**	
Male	48 (61)
Age (years)**	35 [26 - 58]
Age group	
Adults (18–44 years)	51 (64%)
Middle-aged (45–64 years)	14 (18%)
Elderly (≥ 65 years)	14 (18%)
Occupation	
Unemployed / Retired	31 (39%)
Blue-collar worker	8 (10%)
White-collar worker	40 (51%)
**Clinical & Laboratory**	
BMI kg/m²*	24.8 ± 3.8
BMI categories	
Underweight	3 (3.8)
Normal	23 (29)
Overweight	19 (24)
Obesity I Obesity II	10 (13) 24 (30)
Pregnancy	5 (6.3)
Smoking	10 (13)
Diabetes mellitus	11 (14)
Dyslipidemia	43 (54)
Hyperuricemia	17 (22)
**Procedural factors**	
Travel time to the hospital (hour)**	0.58 [0.33, 1.00]
Mode of transportation	
Walking	3 (3.8)
Motorbike	44 (56)
Car	24 (30)
Coach	8 (10)
Monitor application time	
Morning	45 (57)
Afternoon	34 (43)
Waiting time (hour)**	2 [1, 24]
Place of stay during monitoring	
Own home	56 (71)
Relative’s home	8 (10)
Temporary accommodation	15 (19)

*Values are presented as Mean ± SD. **Values are presented as Median [IQR]. *Abbreviations: BMI, Body Mass Index; IQR, Interquartile Range; SD, Standard Deviation*

Compared with ABPM measurements, 30-minute BP values were consistently higher across all comparisons (Table 2). Both SBP and DBP at 30 minutes were significantly higher than 24-hour, daytime, and nighttime ABPM values (p < 0.001). The largest differences were observed when comparing 30-minute BP with nighttime ABPM (SBP: + 12.56 ± 13.06 mmHg; DBP: + 11.92 ± 9.86 mmHg).

**Table 2:** Comparison of 30-minute AOBP and 24-hour ABPM.

Comparison	Mean 30m ± SD	Mean ABPM ± SD	Mean diff ± SD (30m–ABPM)	Paired t (p)	Wilcoxon (p)
**SBP 30m vs 24h**	136.85 ± 13.29	130.27 ± 12.17	+6.59 ± 10.03	p < 0.001	p < 0.001
**DBP 30m vs 24h**	84.73 ± 12.34	77.46 ± 9.36	+7.27 ± 7.47	p < 0.001	p < 0.001
**SBP 30m vs Day**	136.85 ± 13.29	133.13 ± 11.93	+3.73 ± 9.77	0.001	p < 0.001
**DBP 30m vs Day**	84.73 ± 12.34	79.57 ± 9.75	+5.16 ± 7.21	p < 0.001	p < 0.001
**SBP 30m vs Night**	136.85 ± 13.29	124.29 ± 14.54	+12.56 ± 13.06	p < 0.001	p < 0.001
**DBP 30m vs Night**	84.73 ± 12.34	72.81 ± 9.95	+11.92 ± 9.86	p < 0.001	p < 0.001

*Abbreviations: ABPM, Ambulatory Blood Pressure Monitoring; AOBP, Automated Office Blood Pressure; DBP, Diastolic Blood Pressure; SBP, Systolic Blood Pressure; SD, Standard Deviation*

### Bland–altman analysis

Bland–Altman analysis was performed to assess agreement between 30-minute AOBP and ABPM-derived BP values across different time periods (24-hour, daytime, and nighttime), as shown in Figs 2–4.

**Fig 2:**
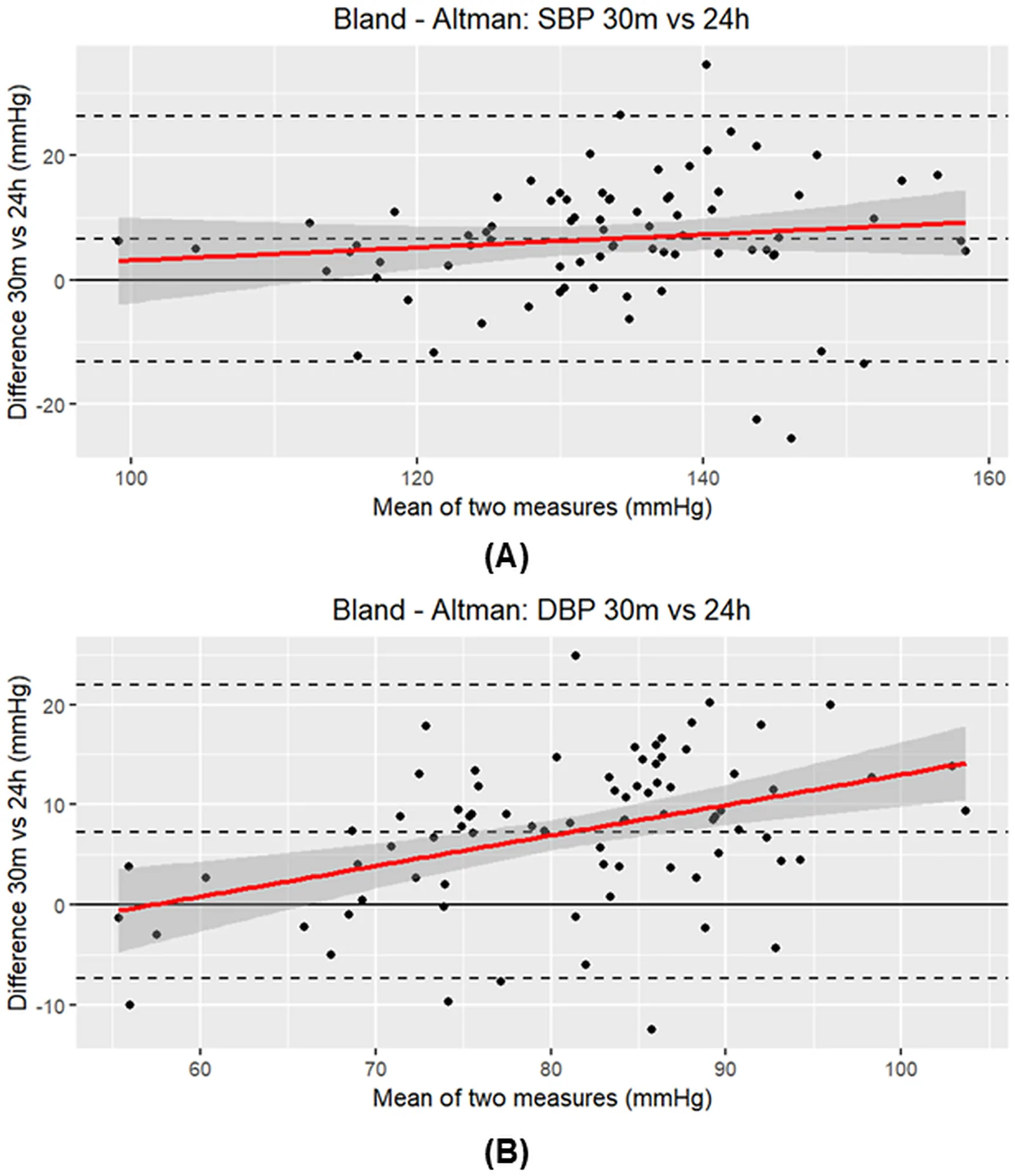
Bland–Altman plots comparing 30-minute AOBP with 24-hour ABPM for systolic and diastolic blood pressure. **(A)** Systolic blood pressure (SBP): The mean bias between 30-minute and 24-hour SBP was + 6.58 mmHg, with limits of agreement from −13.06 to +26.24 mmHg. **(B)** Diastolic blood pressure (DBP): The mean bias between 30-minute and 24-hour DBP was + 7.27 mmHg, with limits of agreement from −7.37 to +21.92 mmHg.

**Fig 3:**
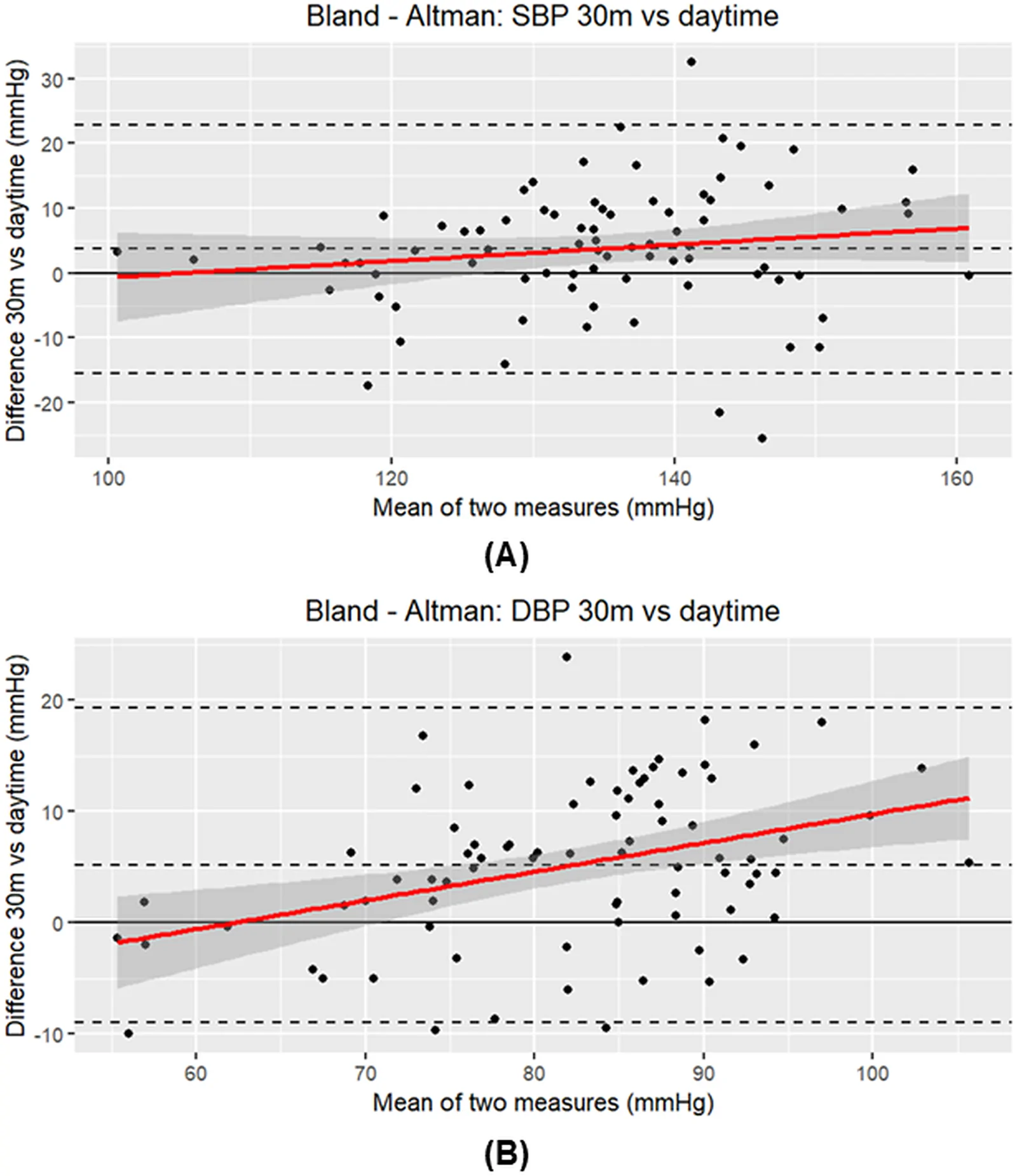
Bland–Altman plots comparing 30-minute AOBP with daytime ABPM for systolic and diastolic blood pressure. **(A)** Systolic blood pressure (SBP): The mean bias between 30-minute and daytime SBP was + 3.72 mmHg, with limits of agreement from −15.43 to +22.88 mmHg. **(B)** Diastolic blood pressure (DBP): The mean bias between 30-minute and daytime DBP was + 5.16 mmHg, with limits of agreement from −8.96 to +19.29 mmHg.

**Fig 4:**
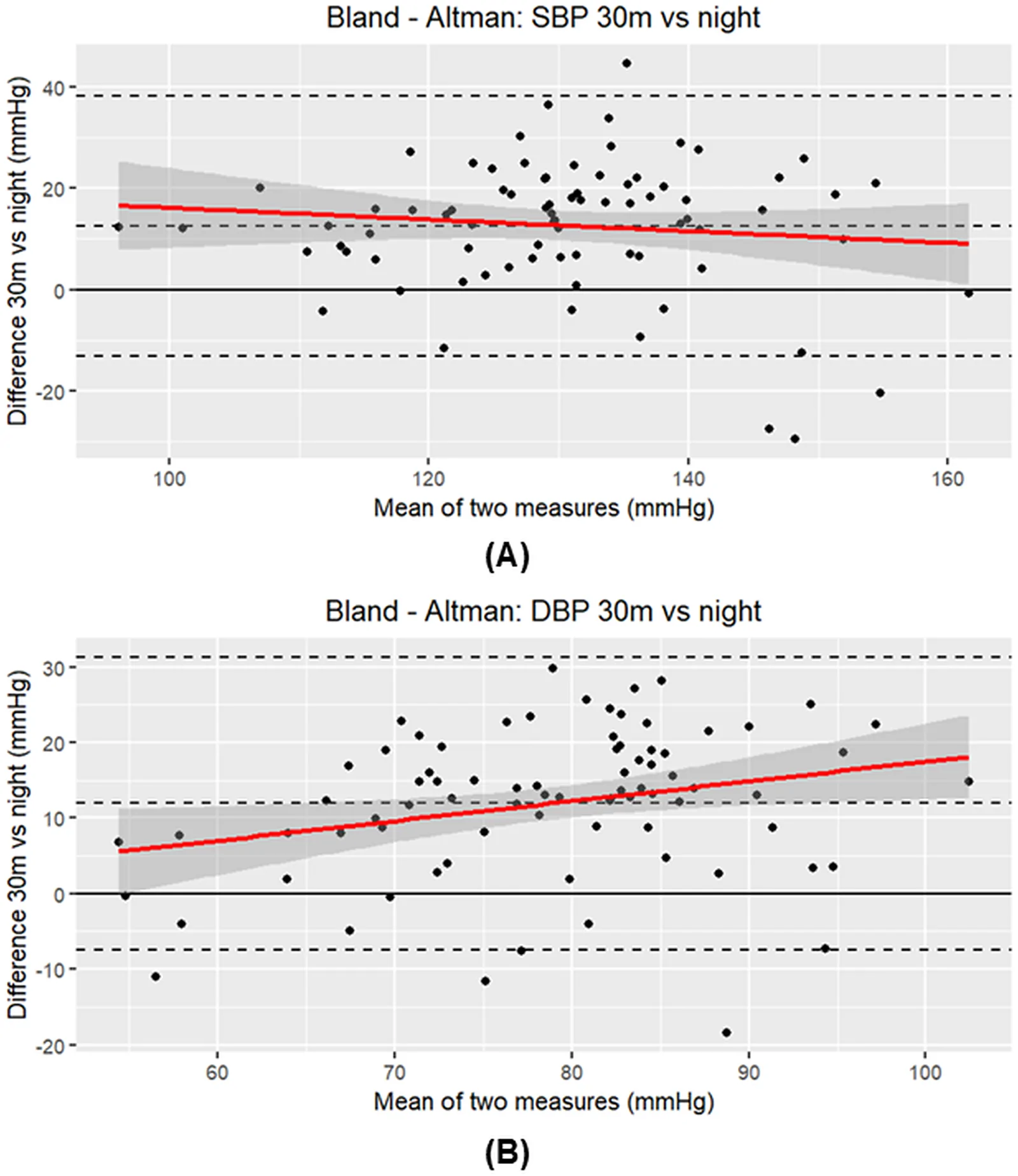
Bland–Altman plots comparing 30-minute AOBP with nighttime ABPM for systolic and diastolic blood pressure. **(A)** Systolic blood pressure (SBP): The mean bias between 30-minute and nighttime SBP was + 12.56 mmHg, with limits of agreement from −13.04 to +38.16 mmHg. **(B)** Diastolic blood pressure (DBP): The mean bias between 30-minute and nighttime DBP was + 11.92 mmHg, with limits of agreement from −7.4 to +31.24 mmHg.

### Diagnostic performance of 30-minute AOBP

Diagnostic performance varied across periods and cut-offs (Table 3). Sensitivity was consistently high (84–96%), whereas specificity ranged from 28% to 79%. For daytime ABPM, the ROC-derived cut-off was ≥ 139/84 mmHg (κ = 0.534), whereas the highest agreement with overall ABPM was observed at ≥140/84 mmHg (κ = 0.624); lower agreement was observed for nighttime ABPM at the ≥ 130/73 mmHg cut-off (κ = 0.284). McNemar’s test indicated significant differences at several cut-offs, particularly for daytime ≥130/80 and overall ≥130/80 (p < 0.05). ROC analysis further demonstrated good discrimination of 30-minute BP measurements against ABPM (Fig 5).

**Table 3:** Diagnostic Agreement Between 30-minute and ABPM (Daytime – Nighttime – Overall – 24-hour).

Period	30-min AOBP cut-off (mmHg)	Se, % (95% CI)	Sp, % (95% CI)	PPV, % (95% CI)	NPV, % (95% CI)	Kappa (p)	McNemar (p)
**Daytime**	≥ 130/80	95 (84-99)	42 (26-59)	66 (53-78)	88 (64-99)	0.378 (p < 0.001)	**p < 0.001**
	≥ 139/84	86 (72-95)	67 (49-81)	76 (61-87)	80 (61-92)	0.534 (p < 0.001)	p = 0.239
**Nighttime**	≥ 130/80	90 (80-96)	61 (36-83)	89 (78-95)	65 (38-86)	0.523 (p < 0.001)	p = 1.000
	≥130/73	95 (86-99)	28 (10-53)	82 (71-90)	62 (24-91)	0.284 (p < 0.001)	**p = 0.024**
**Overall**	≥ 130/80	96 (86-100)	52 (33-71)	77 (65-87)	88 (64-99)	0.523 (p < 0.001)	**p = 0.006**
	≥140/84	84 (71-93)	79 (60-92)	88 (75-95)	74 (55-88)	0.624 (p < 0.001)	p = 0.789
**24 – hour**	≥ 130/80	89 (78-95)	56 (31-78)	87 (76-94)	59 (33-82)	0.45 (p < 0.001)	p = 1.000
	≥130/83	89 (78-95)	67 (41-87)	90 (79-96)	63 (38-84)	0.541 (p < 0.001)	p = 1.000

*Abbreviations: CI, Confidence Interval; NPV, Negative Predictive Value; PPV, Positive Predictive Value; Se, Sensitivity; Sp, Specificity. Kappa assesses diagnostic agreement. p-values are for McNemar’s test.*

**Fig 5:**
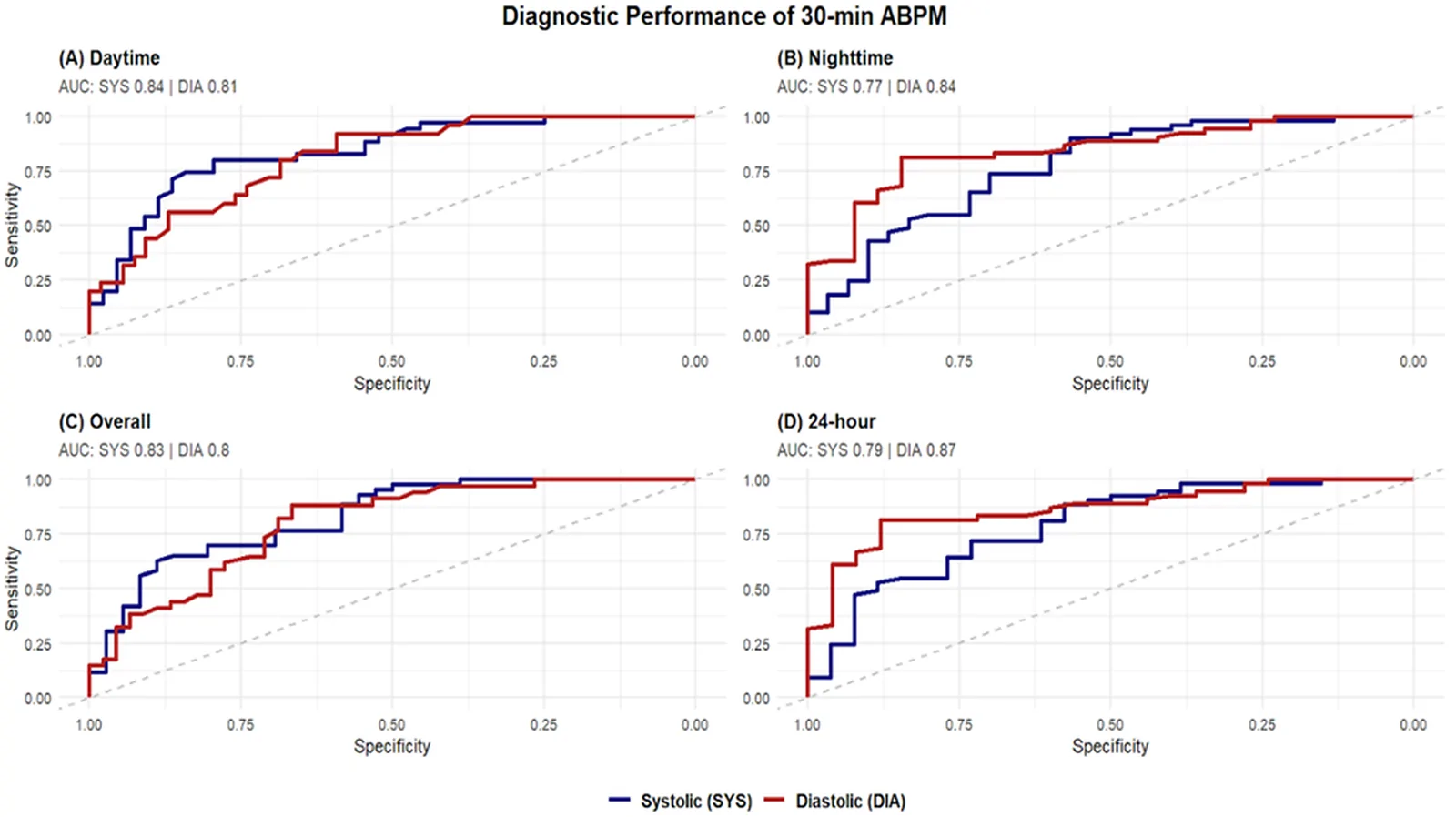
Diagnostic Agreement Between 30-Minute and ABPM (Daytime – Nighttime – Overall – 24-hour). Receiver operating characteristic (ROC) analysis showed good discrimination of 30-min BP against ABPM across all periods. For daytime, AUC was 0.84 (SBP) and 0.81 (DBP); for nighttime, AUC was 0.77 (SBP) and 0.84 (DBP). For the overall reference, AUC was 0.83 (SBP) and 0.80 (DBP). The highest AUC was observed for 24-hour DBP (0.87), while 24-hour SBP showed an AUC of 0.79.

In multivariable logistic regression analysis (Table 4), no factor was significantly associated with diagnostic agreement between 30-minute BP (cut-off ≥130/83 mmHg) and 24-hour ABPM (all p > 0.05). Age showed a borderline association (OR = 0.96, 95% CI: 0.93–1.00; p = 0.06). Sex, occupation, BMI, and waiting time were not independently associated with agreement.

**Table 4:** Multivariable logistic regression analysis of factors associated with diagnostic agreement between 30-minute (Cut-off: ≥ 130/83 mmHg) and 24-hour ABPM.

Characteristic	OR (95% CI)	p – value
Age (per 1-year increase)	0.96 (0.93 - 1)	0.06
Sex		
Female (ref = Male)	3.38 (0.72, 20.9)	0.149
Occupation		
Blue-collar worker (ref = Unemployed)	0.43 (0.05 - 4.07)	0.426
White-collar worker (ref = Unemployed)	1.90 (0.4 - 9.21)	0.412
BMI (kg/m^2^)	0.94 (0.78 - 1.14)	0.513
Waiting time before ABPM	0.99 (0.97 - 1.01)	0.241

*Abbreviation: BMI, Body Mass Index; CI = Confidence Interval, OR = Odds Ratio.*

## Discussion

In this study, 30-minute AOBP was consistently higher than ABPM-derived values across all comparison periods, with mean differences of +6.59/ + 7.27 mmHg versus 24-hour ABPM, + 3.73/ + 5.16 mmHg versus daytime ABPM, and +12.56/ + 11.92 mmHg versus nighttime ABPM for systolic/diastolic BP, respectively. Although Bland-Altman analysis showed that most measurements lay within the limits of agreement, the limits were relatively wide, indicating that agreement was acceptable at the group level but remained imperfect at the individual level. Diagnostic performance analyses further showed high sensitivity but only modest specificity across most thresholds, with the best overall agreement observed at the 30-minute cut-off of ≥140/84 mmHg against overall ABPM (κ = 0.624).

In the study of van der Wel et al. [19], mean 30-minute office BP showed almost no difference from daytime ABPM, with differences of only 0–2 mmHg for systolic and 0–3 mmHg for diastolic BP, and 87% of patients were similarly classified by the two methods. In contrast, our study found a clearer positive bias of 30-minute BP over daytime ABPM, particularly for diastolic BP. The slightly greater diastolic than systolic bias observed in the 24-hour and daytime comparisons may partly reflect residual clinic-related sympathetic activation or differences in peripheral vascular tone during the initial office measurement period, although these mechanisms were not directly assessed in our study and this interpretation remains speculative. Notably, Peeters et al. found that although AOBP and ABPM were significantly correlated, only about 64.7% of systolic and 50.2% of diastolic measurements were within predefined limits of agreement and approximately 73.5% of patients received the same HTN classification [23]. These findings may reflect differences in patient characteristics, measurement environments, the specific 30-minute protocol used, or the timing of office and ambulatory recordings, suggesting that the apparent equivalence between 30-minute AOBP and daytime ABPM may not be universal across clinical settings. Although ROC analysis in our study demonstrated fair-to-good discrimination and consistently high sensitivity across several cut-offs, the relatively wide limits of agreement and modest specificity indicate that 30-minute AOBP may overclassify elevated BP relative to ABPM in a non-negligible proportion of individuals.

The larger discrepancy between 30-minute AOBP and nighttime ABPM in our study was expected. ABPM provides information that office-based approaches cannot capture, particularly nocturnal BP, dipping status, morning surge, and short-term circadian variability. Because nighttime BP is generally lower than awake BP, an awake seated 30-minute office protocol should not be expected to approximate nocturnal values closely. Accordingly, the comparison with nighttime ABPM in our study was exploratory and was intended to assess whether 30-minute AOBP could identify nighttime hypertension. Current reviews and guidelines continue to emphasize that ABPM is preferred when nocturnal HTN, abnormal dipping, resistant HTN, or discrepant office and out-of-office phenotypes are suspected [9,29,30]. The HOPE Asia Network consensus also highlights the importance of out-of-office BP assessment in Asian populations, emphasizing the role of home BP monitoring and unattended automated office BP in detecting phenotypes such as MH, which are important for preventing cardiovascular events [29].

The high sensitivity but only moderate specificity observed in our analysis suggests that 30-minute AOBP may be useful for identifying patients with elevated daytime BP. However, because this method does not capture nighttime BP, normal AOBP values cannot exclude ambulatory HTN, particularly nocturnal HTN. Therefore, confirmation with 24-hour ABPM remains necessary when clinical suspicion persists. These findings align with previous studies showing that automated office techniques reduce white-coat effects compared with routine office measurement, yet still do not completely reproduce ambulatory BP behavior [23]. Recent multicenter data also show that unattended AOBP approaches perform better than standard office BP yet still do not completely eliminate diagnostic misclassification when compared with ABPM [31]. A meta-analysis of 26 studies in 2019 found that AOBP values were substantially lower than physician-measured office BP but not significantly different from daytime ABPM at the group level [32]. Likewise, Haan et al. showed that 30-minute office BP was lower than standardized office BP and had better repeatability, suggesting that serial unattended office measurements may better reflect true BP than conventional office assessment [20].

In our study, the best agreement with overall ABPM was observed at the 30-minute threshold of ≥140/84 mmHg, while lower thresholds such as ≥130/80 mmHg tended to maximize sensitivity at the cost of specificity. This is clinically relevant because no universal threshold for 30-minute AOBP has been established across all protocols. Recent consensus and statements generally consider AOBP values around 135/85 mmHg to correspond to daytime ABPM and home BP thresholds, but they also acknowledge protocol heterogeneity and the need for local validation [29,33]. The most appropriate cut-off may depend on the reference period used, the protocol applied, and the intended clinical purpose, whether screening, ruling out, or confirming HTN.

Our multivariable analysis did not identify independent predictors of diagnostic agreement between 30-minute AOBP and 24-hour ABPM, although age showed a borderline association. Peeters et al. reported that higher BMI and a TIA/stroke cohort were associated with lower agreement in some comparisons, but these associations were not consistent across all models [23].

Some limitations of this study should be acknowledged. The final analyzable sample was smaller than the prespecified target of 95 participants, which may have reduced statistical power to detect subtle differences between measurement methods or identify independent predictors of agreement. In addition, as this was a single-center study, the generalizability of the findings to other populations or clinical settings may be limited. The 30-minute AOBP protocol reflects BP under resting clinical conditions and therefore cannot capture the full circadian variability recorded by 24-hour ABPM. Furthermore, the optimal diagnostic thresholds for 30-minute AOBP are not yet universally standardized, which may influence the observed diagnostic performance. Finally, potential factors affecting BP variability, such as medication timing, daily activity, and sleep quality, were not fully assessed and may have contributed to the disagreement between the two measurement methods.

## Conclusion

Thirty-minute AOBP showed reasonable agreement with daytime ABPM and demonstrated good diagnostic discrimination for identifying elevated ambulatory blood pressure. It may serve as a practical initial assessment tool in clinical settings. However, normal AOBP values cannot exclude ambulatory hypertension, particularly nocturnal HTN, and confirmation with 24-hour ABPM remains necessary when clinical suspicion persists.
