# Supplementary material for: Compare 30-minute automated office blood pressure vs 24-hour ambulatory blood pressure monitoring: A prospective study
Source: PLoS One. 2026 Nov 2;21(11):e0360957. doi: 10.1371/journal.pone.0360957 (PMC13726988; doi:10.1371/journal.pone.0360957)
Supplement: Supplementary file 1 [file pone.0360957.s003.pdf]

Please elaborate. Nice work.

Author: Thank you for this

discrepancies are noted for nighttime values (+12.56 mmHg and +11.92 mmHg for SBP and

reporting guidelines. Specifically, they should

mean nighttime SBP bias of 11.4 mmHg is stated. The authors must verify and synchronize these values.

Author: Thank you for

Author: Thank
