# Supplementary material for: Compare 30-minute automated office blood pressure vs 24-hour ambulatory blood pressure monitoring: A prospective study
Source: PLoS One. 2026 Nov 2;21(11):e0360957. doi: 10.1371/journal.pone.0360957 (PMC13726988; doi:10.1371/journal.pone.0360957)
Supplement: Supplementary file 2 [file pone.0360957.s001.docx]

**1. Main Claims and Significance for the Discipline**

In this single-center study, the authors compare 30-minute Automated Office Blood Pressure (AOBP) measurements with 24-hour Ambulatory Blood Pressure Monitoring (ABPM) values in a cohort of 79 Asian adults. They report good diagnostic discrimination for AOBP in identifying elevated ambulatory BP, alongside a systemic overestimation of BP values relative to the reference standard ABPM method. Crucially, they warn that AOBP cannot rule out ambulatory hypertension (HTN)—particularly nocturnal hypertension—and reflect on the current lack of standardized diagnostic thresholds for defining elevated BP via AOBP measurements.

The results of this study are interesting and clinically significant. Because 30-minute BP values offer a more reliable insight into a patient’s BP profile, this method—demonstrating a reasonable sensitivity of 84% to 96%—may serve as a valuable screening tool in resource-limited settings and high-volume clinics to determine which patients warrant additional 24-hour evaluation.

**2. Contextualization and Fairness to Prior Literature**

The literature is treated fairly and properly contextualized. The authors successfully contrast their findings with studies from Western populations (e.g., van der Wel et al., *Ann Fam Med*, 2011; Peeters et al., *Blood Press*, 2022) and regional consensus guidelines (Shin J et al., The HOPE Asia Network consensus, *J Clin Hypertens*, 2024). Furthermore, they transparently discuss discrepancies with previously reported data.

For instance, while van der Wel et al. reported only marginal differences in daytime systolic and diastolic BP values (with 87% of patients similarly classified by both methods), the current study observes a systemic positive bias for 30-minute AOBP values (+3.72 mmHg for SBP and +5.16 mmHg for DBP daytime values). Even greater discrepancies are noted for nighttime values (+12.56 mmHg and +11.92 mmHg for SBP and DBP, respectively) and 24-hour values (+6.58 mmHg and +7.27 mmHg, respectively).

**3. Data, Statistical Analysis, Support for Claims, and Ethical Considerations**

In the majority of the manuscript, the data and analyses are well presented. However, several sections require critical correction:

- **Ethical Timeline:** On page 8, the authors state that *"upon completion of 30-min AOBP measurement, the investigator approached the patient to provide detailed information about the study and obtain informed consent."* If patient data were recorded prior to obtaining informed consent, this represents an ethical violation. The authors must clarify whether the 30-minute AOBP measurement was performed as standard, routine clinical care, and confirm that consent was obtained prior to using these data for research purposes.
- **Sample Size and Statistical Power:** Based on the authors' power calculation requiring a minimum sample size of 95 patients, the final analyzed sample of 79 patients may be underpowered to detect all relevant differences between the two methods. While this limitation is mentioned in the Discussion, it should also be explicitly stated in the Study Population section that recruitment fell short of the targeted 95 patients due to exclusion criteria.
- **Patient Selection Transparency (STROBE):** The authors must describe the patient selection process more granularly to fully adhere to the STROBE reporting guidelines. Specifically, they should report the exact number of patients who declined inclusion, the exact breakdown of patients meeting each specific exclusion criterion, and the number of recordings discarded due to technical errors or insufficient readings.
- **Missing Data:** The authors should explicitly state whether any variables contained missing data, and explain the statistical methods used to handle those missing values. Also, the authors should provide data whether (1) any of the patients had already been diagnosed with arterial hypertension prior to inclusion in the study, and (2) data regarding antihypertensive drug use.
- **Minor Text and Table Edits:**
  - In the text and figures, the participant age criterion should be consistently formatted as "aged $\geq18$ years".
  - In Table 4, the variable name *"time wait holter"* should be changed to a clearer, more formal academic term, such as *"delay to device initialization"* or *"ABPM device waiting time"*.
- **Data Discrepancies:**
  - On page 12 of the Results section, the authors report a nighttime SBP bias of +12.56 mmHg, which contradicts the Figure 4 caption where a mean nighttime SBP bias of 11.4 mmHg is stated. The authors must verify and synchronize these values.
  - In Table 3, a daytime cut-off value of "$\geq139/84$" is provided without units. Please add "mmHg" and address why this differs from the value of "$\geq140/84$ mmHg" used throughout the main text to ensure internal consistency.

**4. Protocols, Algorithms, and Deviations**

The specific algorithmic steps used for the 30-minute AOBP data collection are described with a sufficient level of detail. Similarly, the data processing protocols and statistical analyses performed are appropriately detailed to allow for thorough understanding.

**5. Data Availability and Software Identification**

The text notes that Microsoft Excel was utilized for data storage and the "R" environment for data analysis. The authors should provide the specific software versions used for both Excel and R. Additionally, the statement that data will be provided upon reasonable request is acceptable.

**6. Adherence to Reporting Guidelines**

As a prospective cohort study, this work must strictly comply with the STROBE guidelines. This requirement extends to the participant sampling workflow illustrated in Figure 1. The authors are kindly requested to formally state their compliance with the STROBE guidelines for reporting observational research within the Methods section.

**7. Reproducibility and Dual Use Research of Concern**

As presented, the methodology is written clearly enough to permit full replication of the clinical experiments by independent investigators. The findings and methodologies detailed in this manuscript do not present any Dual Use Research of Concern.
