# Supplementary material for: Compare 30-minute automated office blood pressure vs 24-hour ambulatory blood pressure monitoring: A prospective study
Source: PLoS One. 2026 Nov 2;21(11):e0360957. doi: 10.1371/journal.pone.0360957 (PMC13726988; doi:10.1371/journal.pone.0360957)
Supplement: Peer review history

# Peer review: Decision Letter 0

**DOI:** 10.1371/journal.pone.0360957.r001

**Authors:** Nataša Marčun Varda

12 Jul 2026

Dear Dr. Nguyen,

Thank you for submitting your manuscript to PLOS ONE. After careful consideration, we feel that it has merit but does not fully meet PLOS ONE’s publication criteria as it currently stands. Therefore, we invite you to submit a revised version of the manuscript that addresses the points raised during the review process.

Please submit your revised manuscript by Aug 26 2026 11:59PM. If you will need more time than this to complete your revisions, please reply to this message or contact the journal office at plosone@plos.org. When you’re ready to submit your revision, log on to https://www.editorialmanager.com/pone/ and select the 'Submissions Needing Revision' folder to locate your manuscript file.

- A letter that responds to each point raised by the academic editor and reviewer(s). You should upload this letter as a separate file labeled 'Response to Reviewers'.
- A marked-up copy of your manuscript that highlights changes made to the original version. You should upload this as a separate file labeled 'Revised Manuscript with Track Changes'.
- An unmarked version of your revised paper without tracked changes. You should upload this as a separate file labeled 'Manuscript'.

If you would like to make changes to your financial disclosure, please include your updated statement in your cover letter. Guidelines for resubmitting your figure files are available below the reviewer comments at the end of this letter.

If applicable, we recommend that you deposit your laboratory protocols in protocols.io to enhance the reproducibility of your results. Protocols.io assigns your protocol its own identifier (DOI) so that it can be cited independently in the future. For instructions see: https://journals.plos.org/plosone/s/submission-guidelines#loc-laboratory-protocols. Additionally, PLOS ONE offers an option for publishing peer-reviewed Lab Protocol articles, which describe protocols hosted on protocols.io. Read more information on sharing protocols at https://plos.org/protocols?utm_medium=editorial-email&utm_source=authorletters&utm_campaign=protocols.

As the corresponding author, your ORCID iD is verified in the submission system and will appear in the published article. PLOS supports the use of ORCID, and we encourage all coauthors to register for an ORCID iD and use it as well. Please encourage your coauthors to verify their ORCID iD within the submission system before final acceptance, as unverified ORCID iDs will not appear in the published article. *Only*  the individual author can complete the verification step; PLOS staff *cannot*  verify ORCID iDs on behalf of authors.

We look forward to receiving your revised manuscript.

Kind regards,

Nataša Marčun Varda, PhD

Academic Editor

PLOS One

Journal Requirements:

When submitting your revision, we need you to address these additional requirements.

1. Please ensure that your manuscript meets PLOS ONE's style requirements, including those for file naming. The PLOS ONE style templates can be found at

https://journals.plos.org/plosone/s/file?id=wjVg/PLOSOne_formatting_sample_main_body.pdf and

https://journals.plos.org/plosone/s/file?id=ba62/PLOSOne_formatting_sample_title_authors_affiliations.pdf.

2. In the online submission form you indicate that your data is not available for proprietary reasons and have provided a contact point for accessing this data. Please note that your current contact point is a co-author on this manuscript. According to our Data Policy, the contact point must not be an author on the manuscript and must be an institutional contact, ideally not an individual. Please revise your data statement to a non-author institutional point of contact, such as a data access or ethics committee, and send this to us via return email. Please also include contact information for the third party organization, and please include the full citation of where the data can be found.

3. If the reviewer comments include a recommendation to cite specific previously published works, please review and evaluate these publications to determine whether they are relevant and should be cited. There is no requirement to cite these works unless the editor has indicated otherwise.

**Comments to the Author**

1. Is the manuscript technically sound, and do the data support the conclusions?

Reviewer #1: Yes

Reviewer #2: Yes

**********

2. Has the statistical analysis been performed appropriately and rigorously?

Reviewer #1: Yes

Reviewer #2: Yes

**********

3. Have the authors made all data underlying the findings in their manuscript fully available?

The PLOS Data policy

Reviewer #1: Yes

Reviewer #2: Yes

**********

4. Is the manuscript presented in an intelligible fashion and written in standard English?

Reviewer #1: Yes

Reviewer #2: Yes

**********

Reviewer #1: The paper accurately present research in comparing 30 minute Automated office BP to 24 hour Ambulatory Blood pressure.

I have a few questions for the authors: If the power was calcualted to be 99 participans, how come your recruitment stopped at 79?

how come the AOBP limit for HTN was different than daytime ABMP? Is there any literature behind that? Can you explain better?

Do you have any explanation why diastolic pressure had higher mean difference?

Is comparing AOBP with nighttime ABPM sensible?

Please elaborate. Nice work.

Reviewer #2: Review

1. Main claims and significance for the discipline:

In a single-center study, the authors compare 30-minute Automated Office BP (AOBP) measurements with 24-hour Ambulatory Blood Pressure Monitoring (ABPM) values in a group of 79 Asian adults and report good discrimination of AOBP in identifying elevated ambulatory BP, yet systemic overestimation of BP values relative to the reference standard ABPM method. Also, the ward that AOBP cannot rule out ambulatory hypertension (HTN), particularly nocturnal hypertension, and reflect on the lack of standardized diagnostic thresholds of AOBP measurements for defining elevated BP values.

The results of the study are interesting and significant for clinical use, as 30-minute BP values offer a more reliable insight into the patients’ BP profile and with a reasonable sensitivity of 84 to 96% may serve as a “screening tool” in resurce-limited settings and high-patient volume clinics for deciding which patients need additional 24-hour evaluation.

2. Contextualization and fairness to prior literature:

The literature is treated fairly and properly contextualized. The authors successfully contrast their findings with studies from Western-population patients (e.g., van der Wel et al. A Novel Approach to Office Blood Pressure Measurement: 30-Minute Office Blood Pressure vs Daytime Ambulatory Blood Pressure. Ann Fam Med. 2011; and Peeters et al. Comparison of automated office blood pressure measurement with 24-hour ambulatory blood pressure measurement. Blood Press. 2022), and regional consensus guidelines (Shin J et al. The HOPE Asia Network consensus on blood pressure measurements corresponding to office measurements: Automated office, home, and ambulatory blood pressures. J Clin Hypertens. 2024). Also, the openly discuss discrepancies with reported findings. For example, van der Wel et al. reported only marginal differences in daytime systolic and diastolic BP values, and 87% of patients in the study by van der Wel were similarly classified by the two methods. In contrast, this study reports a systemic positive bias of 30-minute AOBP values of + 3.7 mmHg in systolic BP and + 5.16 in diastolic BP daytime values, with even greater discrepancies in nigh-time (+12.5 mmHg and + 11.9 mmHg for SBP and DBP, respectively) and 24-hour SBP and DBP values (+ 6.59 mmHg and + 7.27 mmHg, respectively).

3. Data, statistical analysis, support for claims and ethical considerations

In the majority of the manuscript, the data and analysis are well presented. However, some sections require corrections.

First and foremost, on page 8, the authors state that »upon competion of 30-min AOBP measurement, the investigator approached the patient to provide detailed information about the study and obtain informed consent«. If patient data was collected prior to collection of patients' informed consent, this represents a potential ethical violation. Please clarify whether the 30-minute AOBP measurement could be regarded as routine clinical care and whether consent was obtained prior to use of patients data for research puroposes.

Secondly, based on the power calulation of a required sample size of 95 patients, the sample size of 79 patients may not detect all the relevant differences between 30-minute AOBP and 24-hour ABPM measurement. This is noted in the discussion section, but should also clearly be stated in the Study population section, that due to the exclusion criteria, only 79 of the expected 95 patients were enrolled.

Furthermore, the authors should accurately describe the patients selection process. Specifically, adherence to the STROBE reporting guidelines should be maintained, including the reporting of patients who failed to provide informed consent/declined inclusion, patients who met any of the exclusion criteria (and number of patients in each exclusion criteria), patients excluded due to technical errors or insufficient data, the number of patients included in the study, and the number of patients analyzed.

Also, the authors should report if any variables had missing data and how they statistically handled cases with missing data.

In the text and appropriate figures, the age of participants should be described aged ≥ 18 years. A minor text edit from the variable name in Table 4 from »time wait holter« to a clearer term should be made, eg. »delay to device initialization«.

In the Results section, page 12, the authors write that … with nighttime ABPM (SBP + 12.56 mmHg …), a number different to the caption of Figure 4, where a mean bias of nighttime SBP of 11.4 mmHg is reported. Please check whether the appropraite values are reported.

Also, in Table 3, a datime cutoff value of ≥ 139/84 (please provide units mmHg) is provided, which is in contrast to the text-written values of ≥ 140/84 mmHg. Please correct for consitent reporting.

4. Protocols, algorithms and deviations

The specific alrorithmic steps used for 30-minute AOBP data collection is detailed to a sufficient amount. Similarly, the data processing and statistical analyses performed are described in sufficient detail.

5. Data availability and use of software

The text notes that Microsoft Excel was use for data storage and »R« for data analysis. Please provide program versions for Excel and R. Also, data is written to be provided based on a reasonable request, which is sufficient.

6. Adherence to reportinting guidelins

As a prospective cohort study, the STROBE guidelines should be complied to. This was already mentioned regarding Figure 1 on the patient sampling process, and we kindly ask the authors to formally state compliance with the STROBE guidelines for reporting observational studies.

7. Reproducibility of the results, dual use concert

As presented, the methodology is appropriatly writted to allow reproducibility of the results. The presented findings are not of Dual Use concern.

**********

what does this mean?). If published, this will include your full peer review and any attached files.

If you choose “no”, your identity will remain anonymous but your review may still be made public.

**Do you want your identity to be public for this peer review?**  For information about this choice, including consent withdrawal, please see our Privacy Policy

Reviewer #1: No

Reviewer #2: **Yes:**  Tadej Petek

**********

[NOTE: If reviewer comments were submitted as an attachment file, they will be attached to this email and accessible via the submission site. Please log into your account, locate the manuscript record, and check for the action link "View Attachments". If this link does not appear, there are no attachment files.]

To ensure your figures meet our technical requirements, please review our figure guidelines: https://journals.plos.org/plosone/s/figures

You may also use PLOS’s free figure tool, NAAS, to help you prepare publication quality figures: https://journals.plos.org/plosone/s/figures#loc-tools-for-figure-preparation.

NAAS will assess whether your figures meet our technical requirements by comparing each figure against our figure specifications.

Attachment Submitted filename: Review_AOBP.docx

# Author comment: Author response to Decision Letter 1

**DOI:** 10.1371/journal.pone.0360957.r002

10 Sep 2026

Reviewer 1

Reviewer #1: The paper accurately present research in comparing 30 minute Automated office BP to 24 hour Ambulatory Blood pressure.

I have a few questions for the authors: If the power was calcualted to be 99 participans, how come your recruitment stopped at 79?

Author: Thank you for this comment. The calculated minimum sample size was 95 participants. During the predefined study period, 99 participants were enrolled and underwent AOBP and ABPM measurements; however, complete data were available for only 79 participants, as some participants had incomplete data and could not be reached despite repeated contact attempts. As recruitment was limited to the predefined study period, further enrollment was not possible. We acknowledge the smaller-than-planned final sample size as a limitation of the study.

how come the AOBP limit for HTN was different than daytime ABMP? Is there any literature behind that? Can you explain better?

Author: Thank you for this question. Evidence regarding diagnostic thresholds for 30-minute AOBP remains limited. Previous studies have proposed a threshold of 135/85 mmHg, mainly based on comparisons with daytime ABPM. As our primary objective was to evaluate 30-minute AOBP as a potential alternative to 24-hour ABPM, we initially used the same threshold of 130/80 mmHg as 24-hour ABPM. Because diagnostic agreement at this threshold was suboptimal, we subsequently explored AOBP cut-offs that best corresponded to 24-hour, daytime, and nighttime ABPM. These data-derived cut-offs should be considered exploratory. AOBP remains a promising and practical approach to blood pressure assessment, and we hope that further studies will provide sufficient evidence to establish validated AOBP cut-offs corresponding to ABPM thresholds.

Do you have any explanation why diastolic pressure had higher mean difference?

Author: Thank you for this observation. The diastolic bias was slightly greater than the systolic bias in the 24-hour and daytime comparisons, although this pattern was not observed for nighttime BP. Residual clinic-related sympathetic activation or differences in peripheral vascular tone during the initial office measurement period may have contributed to this finding; however, these mechanisms were not directly assessed in our study and this interpretation remains speculative. We have incorporated this explanation into the Discussion.

Is comparing AOBP with nighttime ABPM sensible?

Please elaborate. Nice work.

Author: Thank you for this important point. We agree that 30-minute AOBP and nighttime ABPM are measured under different physiological conditions and are not directly interchangeable. The comparison with nighttime ABPM was included as an exploratory analysis to assess whether 30-minute AOBP could identify nighttime hypertension, rather than to establish equivalence between the two measurements. We have clarified this point in the Discussion and emphasized that 30-minute AOBP cannot replace nighttime ABPM for assessing nocturnal blood pressure or dipping patterns.

Reviewer 2

1. Main Claims and Significance for the Discipline

In this single-center study, the authors compare 30-minute Automated Office Blood Pressure (AOBP) measurements with 24-hour Ambulatory Blood Pressure Monitoring (ABPM) values in a cohort of 79 Asian adults. They report good diagnostic discrimination for AOBP in identifying elevated ambulatory BP, alongside a systemic overestimation of BP values relative to the reference standard ABPM method. Crucially, they warn that AOBP cannot rule out ambulatory hypertension (HTN)—particularly nocturnal hypertension—and reflect on the current lack of standardized diagnostic thresholds for defining elevated BP via AOBP measurements.

The results of this study are interesting and clinically significant. Because 30-minute BP values offer a more reliable insight into a patient’s BP profile, this method—demonstrating a reasonable sensitivity of 84% to 96%—may serve as a valuable screening tool in resource-limited settings and high-volume clinics to determine which patients warrant additional 24-hour evaluation.

2. Contextualization and Fairness to Prior Literature

The literature is treated fairly and properly contextualized. The authors successfully contrast their findings with studies from Western populations (e.g., van der Wel et al., Ann Fam Med, 2011; Peeters et al., Blood Press, 2022) and regional consensus guidelines (Shin J et al., The HOPE Asia Network consensus, J Clin Hypertens, 2024). Furthermore, they transparently discuss discrepancies with previously reported data.

For instance, while van der Wel et al. reported only marginal differences in daytime systolic and diastolic BP values (with 87% of patients similarly classified by both methods), the current study observes a systemic positive bias for 30-minute AOBP values (+3.72 mmHg for SBP and +5.16 mmHg for DBP daytime values). Even greater discrepancies are noted for nighttime values (+12.56 mmHg and +11.92 mmHg for SBP and DBP, respectively) and 24-hour values (+6.58 mmHg and +7.27 mmHg, respectively).

3. Data, Statistical Analysis, Support for Claims, and Ethical Considerations

In the majority of the manuscript, the data and analyses are well presented. However, several sections require critical correction:

Ethical Timeline: On page 8, the authors state that "upon completion of 30-min AOBP measurement, the investigator approached the patient to provide detailed information about the study and obtain informed consent." If patient data were recorded prior to obtaining informed consent, this represents an ethical violation. The authors must clarify whether the 30-minute AOBP measurement was performed as standard, routine clinical care, and confirm that consent was obtained prior to using these data for research purposes.

Author: Thank you for raising this important concern. We apologize for the unclear wording in the original manuscript. Written informed consent was obtained before the 30-minute AOBP measurement and before any research-related data were collected or used (we describe the correct process in Figure 1). The 30-minute AOBP measurements analyzed in this study were therefore obtained only after participants had provided consent. We have revised the Methods to clarify the correct sequence of consent and study procedures.

Sample Size and Statistical Power: Based on the authors' power calculation requiring a minimum sample size of 95 patients, the final analyzed sample of 79 patients may be underpowered to detect all relevant differences between the two methods. While this limitation is mentioned in the Discussion, it should also be explicitly stated in the Study Population section that recruitment fell short of the targeted 95 patients due to exclusion criteria.

Author: Thank you for this comment. We agree that the final analyzable sample was smaller than the prespecified target of 95 participants. During the predefined recruitment period, 99 patients were screened; 20 were excluded because they did not meet the inclusion criteria, had incomplete data, or declined to participate, leaving 79 patients for the final analysis. We have clarified this participant flow in the Sample Size, Sampling, and Data Collection section and have also explicitly acknowledged in the Limitation that the smaller-than-planned sample may have reduced the statistical power of the study.

Patient Selection Transparency (STROBE): The authors must describe the patient selection process more granularly to fully adhere to the STROBE reporting guidelines. Specifically, they should report the exact number of patients who declined inclusion, the exact breakdown of patients meeting each specific exclusion criterion, and the number of recordings discarded due to technical errors or insufficient readings.

Author: Thank you for this important comment. We have revised Figure 1 to provide a more detailed participant flow in accordance with the STROBE reporting guidelines. The revised figure reports the number of patients who declined participation, the number excluded for each specific exclusion criterion, and the number of recordings excluded because of technical errors or insufficient valid readings.

Missing Data: The authors should explicitly state whether any variables contained missing data, and explain the statistical methods used to handle those missing values. Also, the authors should provide data whether (1) any of the patients had already been diagnosed with arterial hypertension prior to inclusion in the study, and (2) data regarding antihypertensive drug use.

Author: Thank you for this important comment. We have clarified the handling of missing data in the Statistical Methods section. No missing values were present among the variables included in the final analysis, and therefore no imputation was required. We have also added information regarding prior arterial hypertension and antihypertensive medication use to the Results. None of the 79 participants had a prior diagnosis of arterial hypertension, and none were receiving antihypertensive medication at the time of study inclusion.

Minor Text and Table Edits:

In the text and figures, the participant age criterion should be consistently formatted as "aged ≥18 years".

Author: Thank you for this comment. We have revised the manuscript and figures to consistently present the age eligibility criterion as “aged ≥18 years.”

In Table 4, the variable name "time wait holter" should be changed to a clearer, more formal academic term, such as "delay to device initialization" or "ABPM device waiting time".

Author: Thank you for this suggestion. We have revised the variable name in Table 4 from “time wait holter” to “Waiting time before ABPM”.

Data Discrepancies:

On page 12 of the Results section, the authors report a nighttime SBP bias of +12.56 mmHg, which contradicts the Figure 4 caption where a mean nighttime SBP bias of 11.4 mmHg is stated. The authors must verify and synchronize these values.

Author: Thank you for identifying this inconsistency. We verified the analysis and confirmed that the correct mean nighttime SBP bias was +12.56 mmHg. We have revised the Figure 4 caption.

In Table 3, a daytime cut-off value of "≥139/84" is provided without units. Please add "mmHg" and address why this differs from the value of "≥140/84 mmHg" used throughout the main text to ensure internal consistency.

Author: Thank you for noting this point. We have added the unit “mmHg” to the cut-off column in Table 3. We also clarify that ≥139/84 mmHg and ≥140/84 mmHg represent different ROC-derived cut-offs for different ABPM reference definitions: ≥139/84 mmHg was identified for comparison with daytime ABPM, whereas ≥140/84 mmHg was identified for overall ABPM and showed the highest agreement (κ = 0.624). We have clarified this distinction in the revised manuscript to avoid the impression that these values are inconsistent.

4. Protocols, Algorithms, and Deviations

The specific algorithmic steps used for the 30-minute AOBP data collection are described with a sufficient level of detail. Similarly, the data processing protocols and statistical analyses performed are appropriately detailed to allow for thorough understanding.

5. Data Availability and Software Identification

The text notes that Microsoft Excel was utilized for data storage and the "R" environment for data analysis. The authors should provide the specific software versions used for both Excel and R. Additionally, the statement that data will be provided upon reasonable request is acceptable.

Author: Thank you for this comment. We have revised the Statistical Methods section to specify the software versions used: Microsoft Excel for Microsoft 365, Version 2608, for data storage and R version 4.6.1 for statistical analysis.

6. Adherence to Reporting Guidelines

As a prospective cohort study, this work must strictly comply with the STROBE guidelines. This requirement extends to the participant sampling workflow illustrated in Figure 1. The authors are kindly requested to formally state their compliance with the STROBE guidelines for reporting observational research within the Methods section.

Author: Thank you for this recommendation. We have added a statement in the Study Design and Setting subsection of the Methods indicating that this prospective cohort study was reported in accordance with the Strengthening the Reporting of Observational Studies in Epidemiology (STROBE) guidelines. We also adjust Figure 1 as noted above.

7. Reproducibility and Dual Use Research of Concern

As presented, the methodology is written clearly enough to permit full replication of the clinical experiments by independent investigators. The findings and methodologies detailed in this manuscript do not present any Dual Use Research of Concern.

Attachment Submitted filename: Potint by point response.docx.pdf

# Peer review: Decision Letter 1

**DOI:** 10.1371/journal.pone.0360957.r003

**Authors:** Nataša Marčun Varda

4 Oct 2026

Compare 30-Minute Automated Office Blood Pressure vs 24-Hour Ambulatory Blood Pressure Monitoring: a prospective study

PONE-D-26-18109R1

Dear Dr. Nghia Phu Nguyen!

We’re pleased to inform you that your manuscript has been judged scientifically suitable for publication and will be formally accepted for publication once it meets all outstanding technical requirements.

Within one week, you’ll receive an e-mail detailing the required amendments. When these have been addressed, you’ll receive a formal acceptance letter and your manuscript will be scheduled for publication.

An invoice will be generated when your article is formally accepted. Please note, if your institution has a publishing partnership with PLOS and your article meets the relevant criteria, all or part of your publication costs will be covered. Please make sure your user information is up-to-date by logging into Editorial Manager at Editorial Manager® and clicking the ‘Update My Information' link at the top of the page. For questions related to billing, please contact billing support.

If your institution or institutions have a press office, please notify them about your upcoming paper to help maximize its impact. If they’ll be preparing press materials, please inform our press team as soon as possible -- no later than 48 hours after receiving the formal acceptance. Your manuscript will remain under strict press embargo until 2 pm Eastern Time on the date of publication. For more information, please contact onepress@plos.org.

Kind regards,

Nataša Marčun Varda, PhD

Academic Editor

PLOS One

Reviewers' comments:

**Comments to the Author**

Reviewer #1: All comments have been addressed

Reviewer #2: All comments have been addressed

**********

2. Is the manuscript technically sound, and do the data support the conclusions?

Reviewer #1: Yes

Reviewer #2: Yes

**********

3. Has the statistical analysis been performed appropriately and rigorously?

Reviewer #1: Yes

Reviewer #2: Yes

**********

4. Have the authors made all data underlying the findings in their manuscript fully available?

The PLOS Data policy

Reviewer #1: Yes

Reviewer #2: Yes

**********

5. Is the manuscript presented in an intelligible fashion and written in standard English?

Reviewer #1: Yes

Reviewer #2: Yes

**********

Reviewer #1: (No Response)

Reviewer #2: I would like to thank the Authors for carefully addressing all of the comments and suggestions raised during the previous review. The revised manuscript has been substantially improved, and the changes made have enhanced its clarity and overall quality. I appreciate the Authors’ thorough and constructive response to the comments and their efforts to improve the manuscript.

**********

what does this mean?). If published, this will include your full peer review and any attached files.

If you choose “no”, your identity will remain anonymous but your review may still be made public.

**Do you want your identity to be public for this peer review?**  For information about this choice, including consent withdrawal, please see our Privacy Policy

Reviewer #1: No

Reviewer #2: **Yes:**  Tadej Petek

**********

# Editor report: Acceptance letter

**DOI:** 10.1371/journal.pone.0360957.r004

**Authors:** Nataša Marčun Varda

PONE-D-26-18109R1

PLOS One

Dear Dr. Nguyen,

I'm pleased to inform you that your manuscript has been deemed suitable for publication in PLOS One. Congratulations! Your manuscript is now being handed over to our production team.

At this stage, our production department will prepare your paper for publication. This includes ensuring the following:

* All references, tables, and figures are properly cited

* All relevant supporting information is included in the manuscript submission,

* There are no issues that prevent the paper from being properly typeset

You will receive further instructions from the production team, including instructions on how to review your proof when it is ready. Please keep in mind that we are working through a large volume of accepted articles, so please give us a few days to review your paper and let you know the next and final steps.

Lastly, if your institution or institutions have a press office, please let them know about your upcoming paper now to help maximize its impact. If they'll be preparing press materials, please inform our press team within the next 48 hours. Your manuscript will remain under strict press embargo until 2 pm Eastern Time on the date of publication. For more information, please contact onepress@plos.org.

You will receive an invoice from PLOS for your publication fee after your manuscript has reached the completed accept phase. If you receive an email requesting payment before acceptance or for any other service, this may be a phishing scheme. Learn how to identify phishing emails and protect your accounts at https://explore.plos.org/phishing.

If we can help with anything else, please email us at customercare@plos.org.

Thank you for submitting your work to PLOS One and supporting open access.

Kind regards,

PLOS One Editorial Office Staff

on behalf of

Prof. Dr. Nataša Marčun Varda

Academic Editor

PLOS One
